# Deep phenotyping of left atrial characteristics: sphericity and stiffness stratify patients with atrial fibrillation and cardioembolic stroke

**DOI:** 10.1007/s10554-025-03510-x

**Published:** 2025-09-18

**Authors:** Amy Clark, James Elhindi, Aaisha Ferkh, Sai Nagaratnam, Luke Stefani, Nina Marty Pangilinan, Katty Duong, Paula Brown, Andrew Duggins, Faraz Pathan, Liza Thomas

**Affiliations:** 1 Department of Cardiology, Westmead Hospital, Westmead, NSW 2145 Australia; 2 Westmead Clinical School, University of Sydney, Camperdown, NSW Australia; 3 WSLHD Research and Education Network, Westmead Hospital, Westmead, NSW Australia; 4 Neurology Department, Westmead Hospital, Westmead, NSW Australia; 5 Charles Perkins Centre, University of Sydney, Nepean Clinical School, Sydney, NSW Australia; 6 Cardiology Department, Nepean Hospital, Sydney, Australia; 7 Southwest clinical school, University of New South Wales, Kensington, NSW Australia

**Keywords:** Cardioembolic stroke, Atrial fibrillation, Echocardiography, Left atrium, Left atrial sphericity, Atrial cardiomyopathy

## Abstract

**Purpose:**

Cardioembolic stroke (CES) comprises a high proportion of ischaemic stroke, and atrial fibrillation (AF) is a major risk factor. Novel left atrial (LA) parameters of stiffness and sphericity, have been posited as markers of atrial cardiomyopathy, that often manifests as stroke and AF. We sought to comprehensively evaluate LA parameters in patients with different grades of AF who suffered CES compared to AF patients without history of stroke.

**Methods:**

Consecutive CES patients who presented to our tertiary institute were prospectively recruited; 155 CES patients were classified by rhythm: sinus rhythm (*n* = 30), paroxysmal AF (PAF, *n* = 88), and permanent AF (perAF, *n* = 37). Patients with AF and no prior stroke (AF-NS) were simultaneously recruited (PAF *n* = 77, perAF *n* = 39). All recruited patients underwent transthoracic echocardiogram with detailed evaluation of LA parameters including volume, function, stiffness, mechanical dispersion, and sphericity.

**Results:**

Increased LA volume, stiffness, sphericity, mechanical dispersion and reduced LA function were observed in both patient groups; parameters worsened as AF grade progressed from PAF to perAF (*p* < 0.05 for all). Additionally, LA function was reduced, while mechanical dispersion, stiffness, and sphericity were increased in CES patients compared to rhythm matched AF-NS patients. Differentiation between AF groups and CES versus AF-NS was achieved through Principle Component Analysis (PCA); the latent factors comprised of LA parameters including LA minimum volume, LA emptying fraction, LA stiffness and circularity (measure of sphericity). We performed receiver operating curves of the parameters identified by PCA analysis; circularity, a measure of LA sphericity, had the highest AUC (0.701, *p* < 0.001) to discriminate CE stroke from AF-NS.

**Conclusion:**

In addition to LA volume and function, novel measures of LA stiffness and sphericity are altered in patients with CES and between grades of AF. Our pilot study has identified that a composite of LA parameters may be important for discriminating increased stroke likelihood in CES patients, and between AF grades. Thus, detailed evaluation of LA parameters may have clinical utility in cardioembolic stroke and AF.

**Graphical abstract:**

Deep phenotyping of left atrial characteristics demonstrates left sphericity and stiffness are important for risk stratification of stroke likelihood in patients with atrial fibrillation.

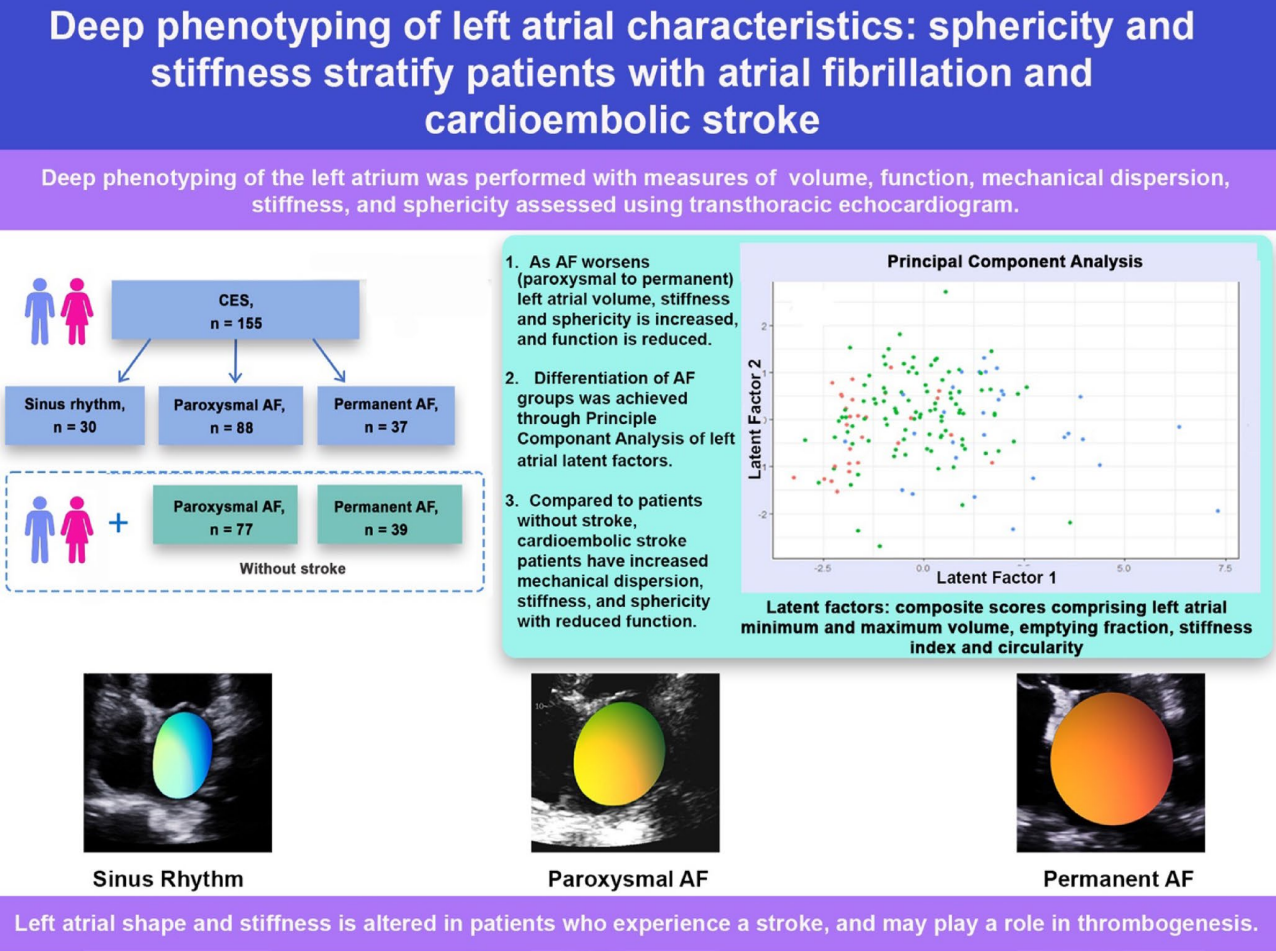

**Supplementary Information:**

The online version contains supplementary material available at 10.1007/s10554-025-03510-x.

## Introduction

Cardioembolic stroke (CES) comprises over one-third of all ischemic strokes and results in higher mortality and disability than other stroke subtypes [1, 2]. The major risk factor for CES is atrial fibrillation (AF) [3]. Currently, ascertaining future stroke risk in AF patients involves CHA_2_DS_2_ -VASC scoring, based exclusively on clinical and demographic characteristics, with anticoagulation recommended for scores ≥ 2 [4]. This model does not however consider other features of AF, notably left atrial (LA) characteristics. A complex relationship has been observed between AF burden and risk of thromboembolism [5]. Adverse left atrial (LA) remodelling has been associated with AF patients compared to control groups in sinus rhythm [6, 7], and with the progression of AF from paroxysmal to permanent, with increased LA volume and reduced function [8].

LA volume and function remodelling have been observed in CES [1, 9], while an independent association has also been observed between LA fibrosis and stroke [10]. Recently, novel transthoracic echocardiogram (TTE) LA parameters have been correlated with LA fibrosis, including LA stiffness [11], mechanical dispersion [12] and LA sphericity [13]. LA sphericity, a determinant of LA shape, has been associated with AF recurrence [14, 15]. However, alterations of these novel LA parameters in CES have not been evaluated. Hence, we sought to (1) comprehensively evaluate novel LA parameters of stiffness, mechanical dispersion, and sphericity in CES patients stratified according to rhythm, and (2) compare LA parameters in CES patients with AF to patients with AF without prior stroke (AF- NS). We hypothesised that (1) novel LA parameters are altered in CES patients, and (2) deep phenotyping of the left atrium, encompassing novel LA parameters may demonstrate significantly worse LA characteristics in patients with AF who suffer CES compared to AF-NS patients.

## Methods

### Study population

**Fig. 1:**
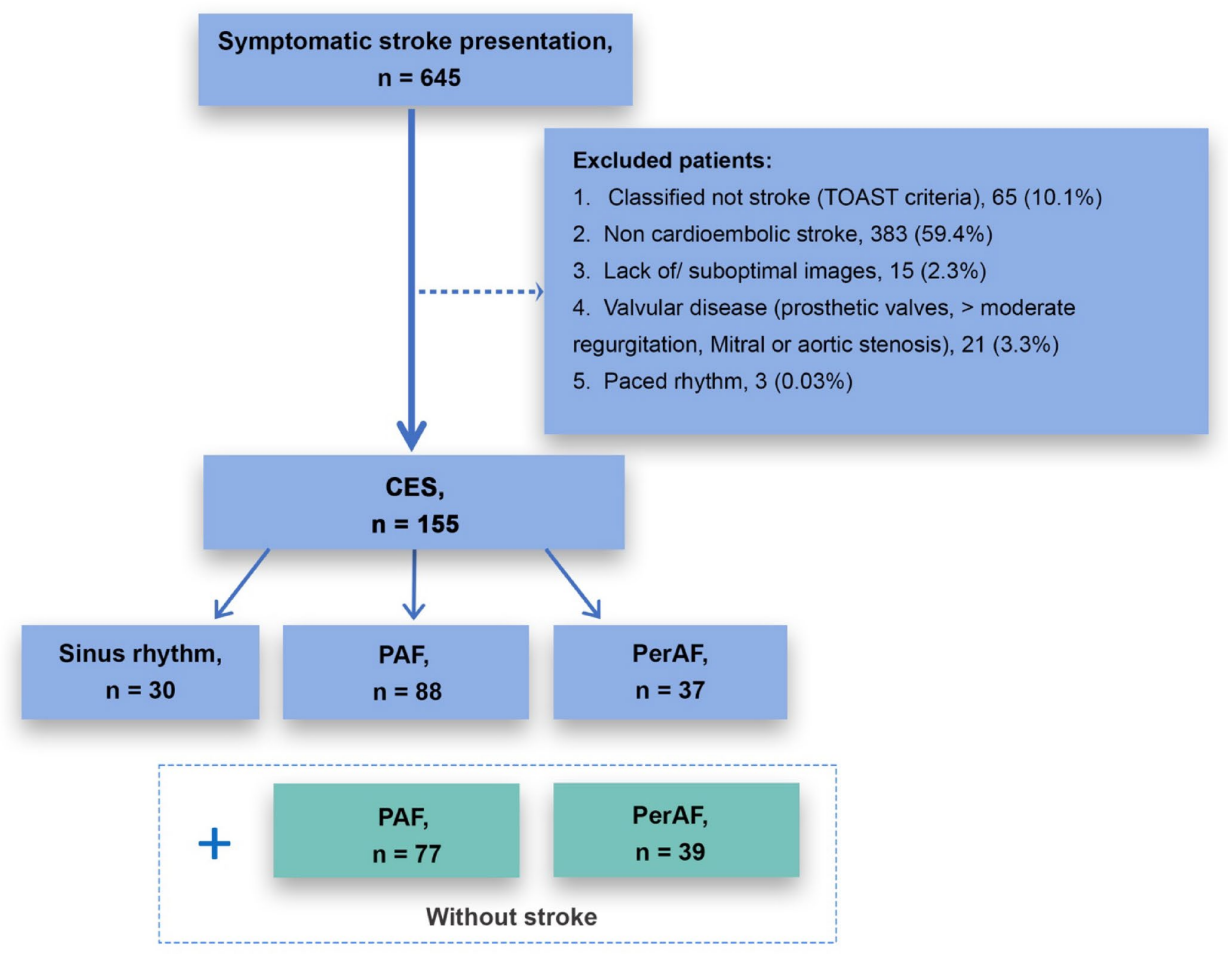
Patient recruitment pathway. CES: cardioembolic stroke, PAF: paroxysmal atrial fibrillation, perAF: permanent atrial fibrillation

In this ‘real world’ study, consecutive patients with a clinical diagnosis of CES were prospectively enrolled (March 2017 - February 2023) at Westmead Hospital, Sydney, Australia. Patients > 18 years were classified according to TOAST (Trial of Org 10172 in Acute Stroke Treatment) criteria [16] by 2 independent neurologists (study inclusion Fig. 1). Patients presenting to hospital with primary AF [17] with no precipitating cause (e.g. myocardial ischemia, heart failure or significant valvular disease (greater than moderate)), and no documented prior stroke history (AF-NS) were simultaneously recruited (March 2017 -December 2021). Patients were adjudicated for AF during hospital admission using electrocardiograms, telemetry (minimum 24 h) and review of medical history. Patients were classified as having sinus rhythm, paroxysmal AF (PAF) < 7 days, or permanent AF (perAF) > 7 days [18]. Clinical and demographic data were obtained including cardiovascular risk factors, and comprehensive TTE evaluation was performed. Clinical risk scores for stroke (CHA_2_DS_2_-VASc) and AF (CHARGE-AF: *C*ohorts for *H*eart and *A*ging *R*esearch in *G*enomic *E*pidemiology-AF) were calculated. Informed consent was obtained from patients with study approval from the Research Ethics Committee of the Western Sydney Local Health District (HREC reference: LNR/16/WMEAD/458).

### Echocardiography

TTE examinations were performed by experienced sonographers using commercially available ultrasound systems (Vivid E95, GE Healthcare), a 3.5 MHz transducer, with optimised LA images acquired at high frame rate (> 55fps). Offline strain analysis was performed by EchoPac Q-analysis (General Electric EchoPac v204 (Milwaukee, WI, USA)) by investigators blinded to patient clinical history. TTE measurements were performed according to current recommendations (detailed description Supplementary Data, Table S1) [19, 20].

### Left atrial assessment

**Fig. 2:**
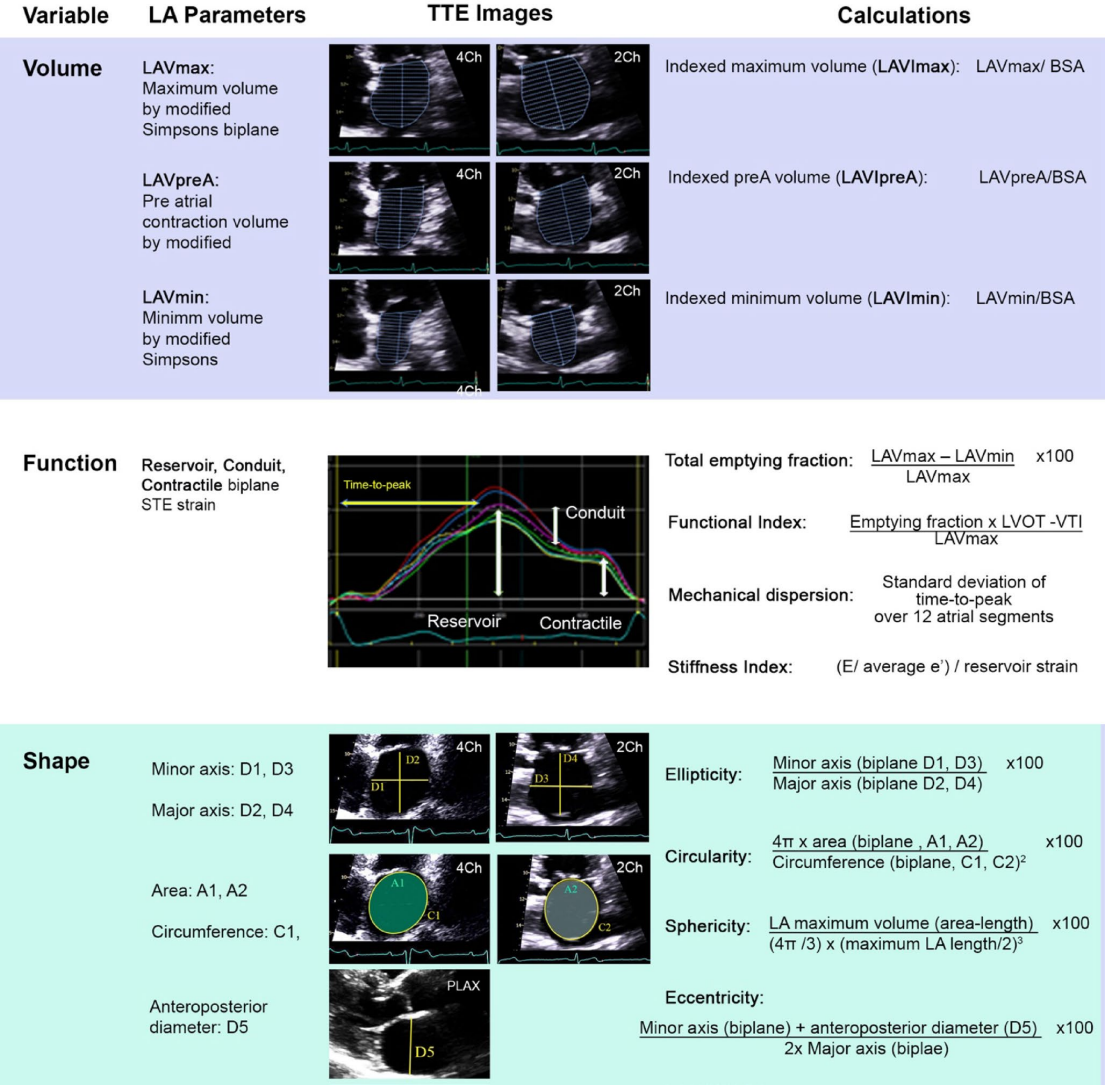
Comprehensive transthoracic echocardiogram assessment of left atrial volume, function, and shape. BSA: body surface area. LA: left atrial. TTE: transthoracic echocardiogram

Using LA optimised TTE views, LA volume, function [20, 21] and novel measures of stiffness, mechanical dispersion [22], and sphericity [13] were evaluated (Fig. 2). LA strain was assessed using speckle tracking of apical 4- and 2- chamber views, using six segments from each view, with R-R gating of the cardiac cycle. The peak positive systolic strain was measured (reservoir strain) [23]. Subsequent decrease in LA strain with a plateau in the strain curve in early diastole following following mitral valve opening (conduit strain), and the second smaller wave in late diastole following active atrial contraction (contractile strain) were not assessed as the majority of patients were in AF at the time of echocardiogram.

Left atrial stiffness index was assessed using a ratio of E/E’ to LA reservoir strain. Mechanical dispersion was calculated using the standard deviation of time-to-peak reservoir strain across 12 atrial segments. As chamber sphericity is a novel parameter, this was assessed using 4 previously defined 2D sphericity techniques [13] including:

1. Ellipticity – biplane average of minor/major LA axis x100.
2. Circularity – ratio of apical biplane LA area to LA circumference x100.
3. Sphericity index – ratio of maximum biplane LA volume (area-length method) to LA volume of a sphere, calculated with largest diameter x100.
4. Eccentricity – ratio of biplane major axis (squared) to the sum of minor axis and anteroposterior diameter x100.

### Statistical analysis

Statistical analysis was performed using IBM SPSS Statistics version 29 (Chicago, Il, USA) and R Studio version 4 (Vienna, Austria). Categorical data are presented as counts (percentage) and compared using $\:\chi\:$^2^ test or Fisher’s exact test. Continuous variables are presented as mean ± standard deviation or median (interquartile range). Normally distributed groups were compared using independent Students *t-* test or one-way analysis of variance (ANOVA) with Bonferroni correction. Non-normally distributed groups were compared using Mann-Whitney U test or Kruskal-Wallis one-way analysis of variance.

We further evaluated which LA parameters showed the highest discrimination between AF groups in both stroke and AF-NS cohorts. Univariate and multivariate multinomial logistic regression models with an omnibus Wald test, and collinearity assessment were used to determine plausibly relevant LA parameters. Measurements were grouped according to concepts of sphericity, function, volume, stiffness and mechanical dispersion, to ensure a balance of statistical and clinical appropriateness (Supplementary Data, Table S3). Principal component analysis (PCA) was performed to observe similarities and differences within the AF groups based on latent factors (LF), using composite scores of weighted LA parameters. PCA helps identify the most important factors that determine variation when several parameters are being compared. Due to the presence of multiple correlated and interrelated LA parameters, we performed PCA to determine the most influential LA parameters in AF discrimination within stroke patients, as well as between CES and AF-NS cohorts. PCA models with highest variance were reported for grades of AF and CES and AF-NS groups. PCA data was presented graphically in score-plots with visual assessment for trends. Factor loadings were discussed wholistically, so a significance level was not defined.

Receiver operating curve (ROC) analysis was performed to determine the area under the curve (AUC) for the various plausible LA parameters identified from PCA, to determine the accuracy of the particular LA parameter to differentiate between CES-AF and NS-AF groups. Pairwise comparison of ROC was performed using Delong test.

### Reproducibility

Intra- and interobserver variability was assessed using intraclass correlation and coefficient of variation (CV) in 15 randomly selected CES patients for LA volume, strain and sphericity, repeated > 1 month following initial assessment by the same investigator and second independent investigator blinded to original results. Intraclass correlation coefficient (ICC) estimates with 95% confidence intervals were calculated based on an absolute-agreement, 2-way mixed-effects model.

## Results

### Patient characteristics

**Fig. 3:**
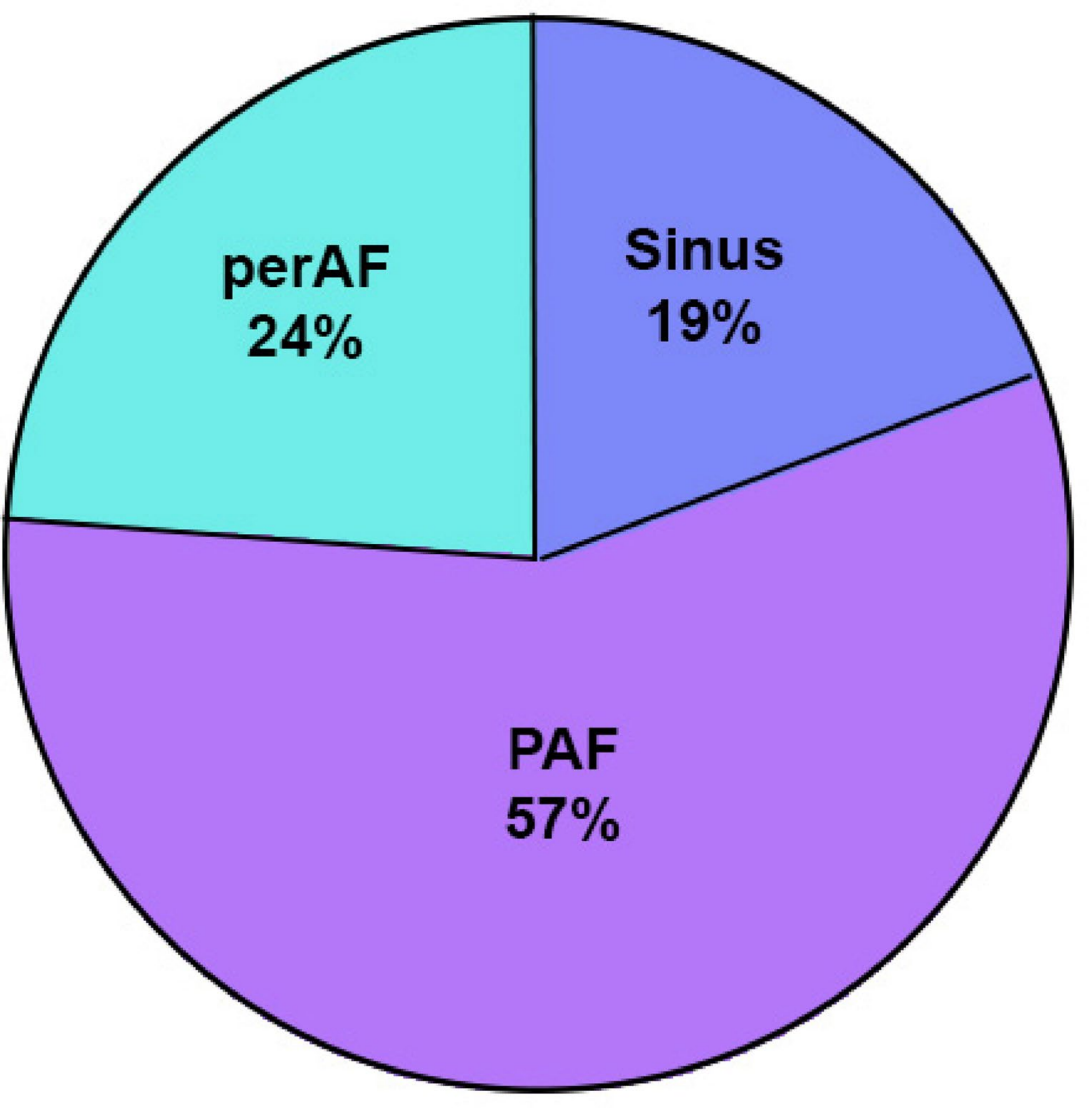
Level of AF in cardioembolic stroke patients. AF: atrial fibrillation, PAF: paroxysmal AF, perAF: permanent AF

From 645 consecutive stroke patients who consented to participate in this study, 155 (24%) CES patients were eligible for study inclusion (Fig. 1); 30(19.4%) patients were in sinus rhythm, 88(56.8%) in PAF, and 37(23.9%) in perAF (Fig. 3). CES in sinus rhythm predominantly occurred due to patent foramen ovale (PFO) without other identifiable cause (*n* = 20, 66.7%) (full summary Supplementary Data Table S2).

**Fig. 4:**
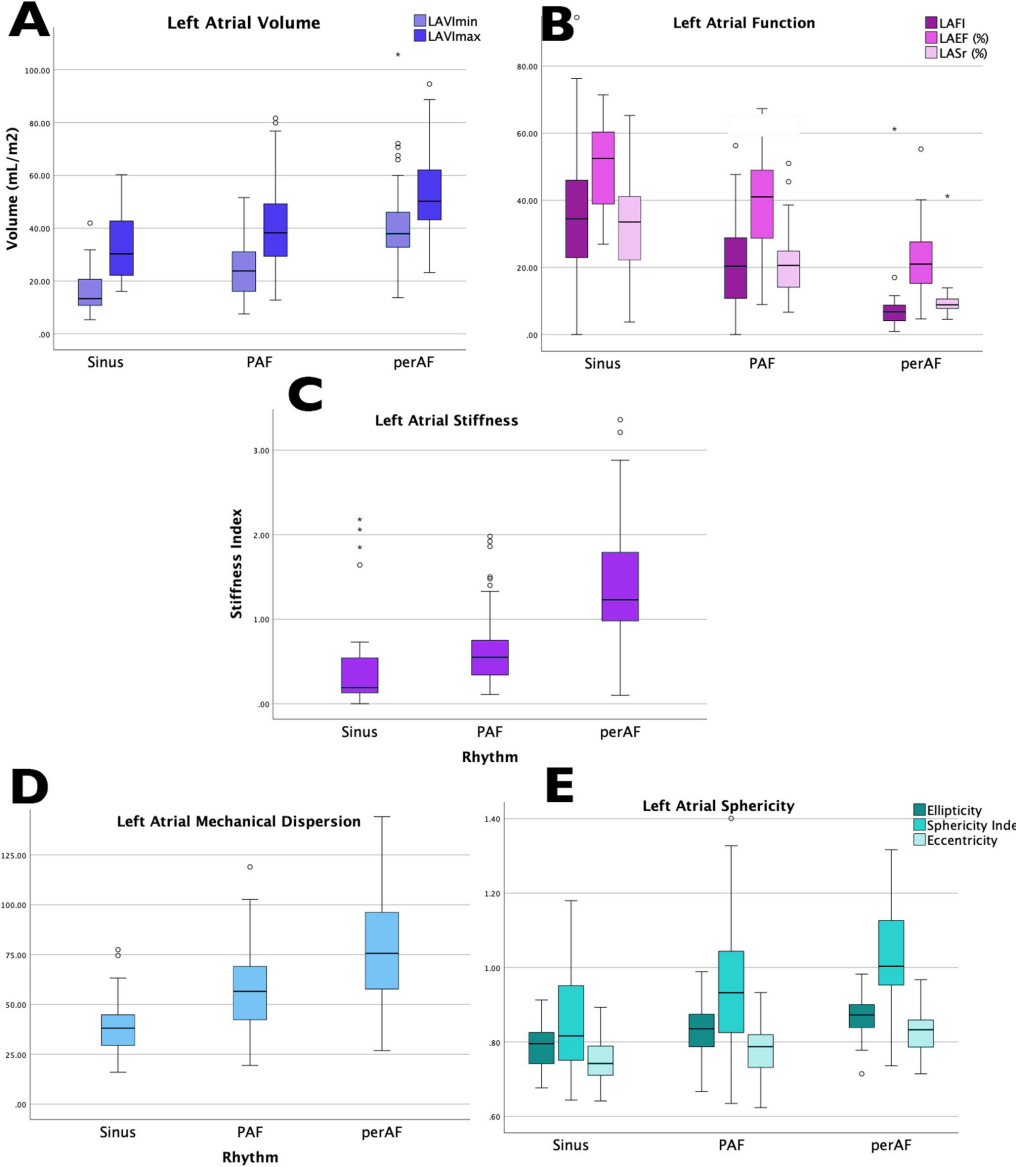
Fig. 4 LA changes in cardioembolic stroke patients with increasing level of atrial fibrillation. A. Minimum (LAVImin) and maximum (LAVImax) indexed volumes. B. Functional index (LAFI), emptying fraction (LAEF), reservoir strain (LASr). C. Ellipticity, sphericity index, and eccentricity. D Stiffness index. E Mechanical Dispersion. LA: left atrial, PAF: paroxysmal atrial fibrillation, perAF: permanent atrial fibrillation, Sinus: sinus rhythm

Baseline characteristics of CES patients are summarised based on rhythm (Table 1). Patients in sinus rhythm were younger (53 years ± 18) than both PAF and perAF patients (*p* < 0.001 for both). Both AF groups had more cardiovascular risk factors, with significantly higher CHA_2_DS_2_-VASc and CHARGE-AF scores. Prior stroke was ascertained in 26 (30%) PAF patients and 13 (36%) perAF patients. In patients with PAF, newly identified AF was observed at presentation with stroke in 52 (34%) patients, corresponding with a lower use of anticoagulation (17% PAF, 60% perAF) on admission in this group.

**Table 1:** Baseline clinical characteristics for all CES patients according to underlying rhythm

	Sinus rhythm *n* = 30	PAF *n* = 88	PerAF *n* = 37	*p* value
*Clinical parameters*				
Age, years	53 ± 18^*†^	76 ± 12	77 ± 12	< 0.001
Sex, male	22(73)^†^	51(58)	19(51)	0.123
Heart rate, bpm	71 ± 12	72 ± 13	73 ± 15	0.820
Systolic BP, mmHg	128 ± 18^*^	137 ± 18^§^	130 ± 19	0.030
Diastolic BP, mmHg	78 ± 12	73 ± 11	73 ± 12	0.174
BSA, m^2^	1.87 ± 0.24	1.86 ± 0.25	1.79 ± 0.27	0.368
CHA_2_DS_2_-VASc	1.40 ± 1.61^*†^	3.61 ± 2.04	4.14 ± 1.92	< 0.001
CHARGE-AF	10.01 ± 1.67^*†^	12.19 ± 1.19	12.01 ± 1.17	< 0.001
*Comorbidities*				
Hypertension	13(43)^*†^	61(70)^§^	34(94)	< 0.001
Hypercholesterolaemia	10(33) ^*†^	56(64)	23(64)	0.009
Diabetes	6(20)	24(28)	9(25)	0.711
Ischaemic heart disease	3(10)^†^	18(21)	11(31)	0.123
Heart failure	2(7)	6(7)^§^	7(19)	0.088
Previous CVA/TIA	2(7)^*†^	26(30)	13 (36)	0.017
*Pharmacotherapy*				
Anticoagulation	0(0)^*†^	15(17)^§^	22(60)	< 0.001
Antiplatelet	5(16.7)	26(30.6)	11(29.7)	0.324
Statin	5(16.7)^*†^	47(54.7)	21(58.4)	< 0.001
ß-blocker	3(10.0)^†^	20(23.3)^§^	16(44.4)	0.005
ACE/ARB	8(26.7)^†^	39(44.8)^§^	25(69.4)	0.002

Data presented as mean ± SD, median (IQR), or number (%). *ACE*: angiotensin-converting enzyme, *AF*: atrial fibrillation, *ARB*: angiotensin receptor blocker, *BP*: blood pressure, *bpm*: beats per minute, *BSA*: body surface area, *CES*: Cardioembolic stroke, *CVA*: cerebrovascular accident, *PAF*: paroxysmal AF, *perAF*: permanent AF, *RV*: right ventricle, *TIA *transient ischaemic attack

*P < 0.05 versus PAF, ^†^P < 0.05 versus peraf, ^§^P < 0.05 versus PerAF

### Echocardiographic LV parameters

TTE demonstrated larger end-diastolic LV volume (LVEDVi, p < 0.001) and lower LV filling pressure estimated by E/e’ (*p* < 0.001) in CES patients in sinus rhythm (Table 2). In contrast, perAF patients had the largest LV wall thickness (*p* < 0.001) and lowest GLS (*p* < 0.001). As AF burden increased from sinus rhythm to PAF and perAF, a significant decrease in LV GLS and an increase in E/e’ was observed (*p* < 0.001).

**Table 2:** Baseline TTE parameters for CES patients based on rhythm

	Sinus rhythm *n* = 30	PAF *n* = 88	PerAF *n* = 37	*p* value
*Standard TTE measurements*				
LVEDVi, ml/m2	53.77 ± 18.6^*†^	44.55 ± 13.99^§^	36.53 ± 13.33	< 0.001
LVESVi, ml/m2	24.23 ± 18.41^*†^	18.17 ± 8.21	15.01 ± 6.83	0.002
Avg wall thickness, mm	10.0 ± 2.5^†^	10.4 ± 1.8^§^	12.0 ± 2.2	< 0.001
LVMi, g/m2	88.4 ± 33.9	88.5 ± 26.4^§^	99.3 ± 27.5	0.128
LVEF, %	58.3 ± 15.0	59.9 ± 9.1	59.5 ± 10.3	0.761
LVGLS, %	−18.96 ± 5.27^†^	−17.64 ± 3.82^§^	−15.09 ± 4.38	< 0.001
Peak E	64.2 ± 14.4^†^	74.9 ± 23.8	86.1 ± 28.7	0.003
Peak A	58.9 ± 21.2^*^	74.5 ± 26.9		0.023
E/A ratio	1.16(0.81–1.58)	0.82(0.68–1.31)		0.161
Deceleration time	205 ± 52	223 ± 72^§^	180 ± 65	< 0.001
E’	9.05 ± 3.86^*†^	6.81 ± 2.05	7.36 ± 2.07	< 0.001
E/E’	6.7(4.70–11.04)^*†^	10.55(8.42–13.71)^§^	12.36(9.83–15.1)	< 0.001
*LA parameters*				
LAVImin, ml	16.4 ± 9.0^*†^	24.8 ± 10.9^§^	44.0 ± 22.3	< 0.001
LAVImax, ml	31.5 ± 11.7^†^	39.9 ± 14.5^§^	56.1 ± 23.9	< 0.001
LAEF	49.8 ± 12.8^*†^	38.2 ± 13.8^§^	22.7 ± 10.3	< 0.001
LA function index	34.5(20.7–46.1)^*†^	20.1(10.1–28.8)^§^	6.7(4.1–9.0)	< 0.001
Reservoir strain	31.84 ± 14.83^*†^	21.00 ± 9.24^§^	10.04 ± 5.87	< 0.001
Mechanical dispersion	40.0 ± 14.8^*†^	56.7 ± 19.3^§^	78.9 ± 25.9	< 0.001
Stiffness index	0.19(0.13–0.70)^*†^	0.55(0.35–0.81)^§^	1.23(0.97–1.81)	< 0.001
Ellipticity	79.1 ± 6.5^*†^	88.4 ± 6.4^§^	91.3 ± 5.3	< 0.001
Circularity	89.4 ± 2.1^*†^	91.1 ± 2.0^§^	92.1 ± 1.8	< 0.001
Sphericity index	50.0 ± 12.6^*†^	70.5 ± 13.4^§^	76.9 ± 13.0	< 0.001
Eccentricity	75.3 ± 5.9^†^	86.7 ± 6.9^§^	90.7 ± 5.	< 0.001

^*^P < 0.05 versus PAF, ^†^*P* < 0.05 versus perAF, ^§^*P* < 0.05 versus perAF

*AF*: atrial fibrillation, *CES*: cardioembolic stroke, *E’*: E prime, *GLS*: global longitudinal strain, *LA*: left atrial, *LAEF*: LA emptying fraction, *LAVImax*: maximum LA volume indexed, *LAVImin*: minimum LA volume indexed, *LVEDVi*: end-diastolic volume indexed, *LVEF:* ejection fraction, *LVMi*: left ventricular mass indexed, *LVESVi*: end-systolic volume indexed, *PAF*: paroxysmal AF, *perAF*: permanent AF, *RV*: right ventricle, *TTE*: transthoracic echocardiogram

### Echocardiographic LA parameters

LA remodelling was observed in CES patients with LA dilatation (Indexed LA maximum volume, (LAVImax) > 34 ml/m2) [20] observed in 37% of patients in sinus rhythm, 60% PAF patients, and 89% perAF patients. From sinus rhythm to PAF to perAF, a progressive increase was observed in maximum and minimum LA volumes (Table 2; Fig. 4a). Conversely as AF level increased, a progressive reduction in LA function was observed in emptying fraction (p < 0001), reservoir strain (p < 0.001), and LAFI (p < 0.001) (Fig. 4b). Of the novel LA parameters, LA stiffness (Fig. 4c) and mechanical dispersion (Fig. 4d) were increased (*p* < 0.001 for all). A similar increase was noted in all measures of LA sphericity including ellipticity, circularity and sphericity index (*p* < 0.001 for all) (Fig. 4e).

**Fig. 5:**
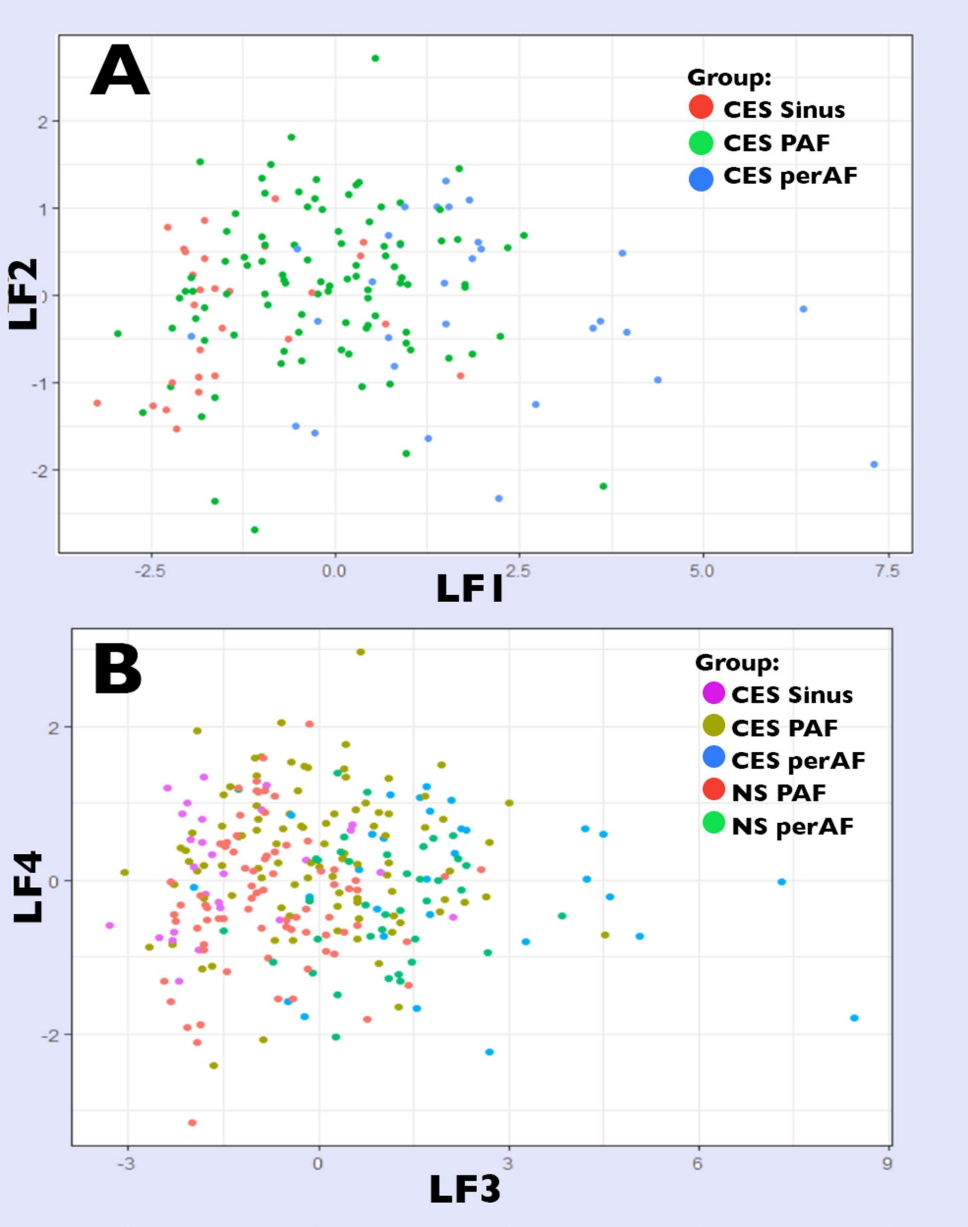
Principal Component Analysis (PCA) score-plots showing distribution of patients according to level of AF. (**A**) PCA1 score-plot of CES patients using LF1 and LF2 derived from LA components. (**B**) PCA2 score-plot of CES and NS-AF patients using LF3 and LF4 derived from LA components. AF: atrial fibrillation, CES: cardioembolic stroke, LA: Left atrial, LF: latent factor, LV: left ventricular

### AF-CES vs. AF-NS

Comparison of all AF-associated CES patients with AF-NS patients (Table 3) showed CES patients were older (*p* < 0.001) with higher rates of comorbidities including hypertension (*p* = 0.023), hypercholesterolaemia, (*p* = 0.001),and diabetes (*p* = 0.035), with correspondingly higher CHA_2_DS_2_-VASc score (*p* < 0.001). AF-NS patients were more commonly male (*p* = 0.036) with a higher CHARGE-AF score (*p* = 0.003), and larger left ventricle (*p* = 0.002). In CES patients with AF, significant alterations in LA parameters was noted; larger LA volumes with reduced LA function, and higher measures of mechanical dispersion, stiffness index, and sphericity compared to patients with AF-NS.

**Table 3:** Baseline comparison of AF patients (AF-associated CES patients and AF-NS patients)

	CES-AF, *n* = 125	AF-NS, *n* = 116	*p* value
*Clinical parameters*			
Age, years	76 ± 12	68 ± 9	< 0.001*
Sex, male	70(56)	79(68)	0.036*
BSA, m^2^	1.84 ± 0.26	1.95 ± 0.23	< 0.001*
CHA_2_DS_2_-VASc	3.77 ± 2.01	2.41 ± 1.32	< 0.001*
CHARGE-AF	12.13 ± 1.19	12.56 ± 1.05	0.003*
*Comorbidities*			
Hypertension	95(77)	75(65)	0.023*
Hypercholesterolaemia	79(64)	51(44)	0.001*
Diabetes	33(27)	19(16)	0.035*
IHD	29(24)	14(26)	0.437
Heart failure	13(11)	9(8)	0.293
Previous CVA/TIA	39(32)	0(0)	< 0.001*
*Standard TTE*			
LVEDVi, ml/m2	42.16 ± 14.23	47.80 ± 15.34	0.003*
LVESVi, ml/m2	17.23 ± 7.93	22.14 ± 11.12	< 0.001*
Wall thickness, mm	10.8 ± 2.1	10.4 ± 1.8	0.072
LVMi, g/m2	91.7 ± 27.1	95.3 ± 24.9	0.288
LVEF, %	59.8 ± 8.8	54.4 ± 11.9	< 0.001*
LVGLS, %	−16.9 ± 4.1	−16.9 ± 4.7	0.891
*LA parameters*			
LAVImin, ml	30.8 ± 17.6	26.7 ± 12.1	0.037*
LAVImax, ml	44.3 ± 19.6	38.5 ± 13.0	0.008*
Total EF	33.2 ± 14.3	32.4 ± 14.2	0.646
LA function index	18.6(7.0-27.8)	20.5 ± 16.4	0.359
Reservoir strain	17.8 ± 9.8	21.3 ± 10.2	0.009*
Mechanical dispersion	63.4 ± 23.7	47.6 ± 15.7	< 0.001*
Stiffness index	0.69(0.41–1.18)	0.49(0.35–0.83)	0.010*
Ellipticity	89.3 ± 6.2	84.6 ± 6.7	< 0.001*
Circularity	91.4 ± 2.0	89.8 ± 2.3	< 0.001*
Sphericity index	72.4 ± 13.5	60.3 ± 13.9	< 0.001*
Eccentricity	87.8 ± 6.8	83.1 ± 6.8	< 0.001*

*P<0.05. AF: atrial fibrillation, *CES*: cardioembolic stroke, *E’*: E prime, *GLS*: global longitudinal strain, *LA*: left atrial, *LAEF*: LA emptying fraction, *LAVImax*: maximum LA volume indexed, *LAVImin*: minimum LA volume indexed, *LVEDVi*: end-diastolic volume indexed, *LVEF*, ejection fraction, *LVMi*: left ventricular mass indexed, *LVESVi*: end-systolic volume indexed, *NS*: no stroke; *PAF*: paroxysmal AF, *perAF*: persistent AF, *RV*: right ventricle, *TTE*: transthoracic echocardiogram

### PAF TTE parameters

Comparison of TTE parameters in PAF patients (CES versus AF-NS) (Table 4) demonstrated increased LA maximum and minimum volumes, reduced function and increased mechanical dispersion and stiffness, in CES patients compared to AF-NS patients. LA sphericity demonstrated larger ellipticity, circularity, sphericity index and eccentricity in CES-PAF patients. LV parameters were similar in both groups.

**Table 4:** Comparison of TTE measurements between CES versus AF-NS patients for grades of AF

	CES PAF, *n* = 88	AF-NS PAF, *n* = 77	*p* value	CES perAF, *n* = 37	AF-NS perAF, *n* = 39	*p* value
Age	76 ± 12	68 ± 9	< 0.001	77 ± 12	68 ± 8	< 0.001*
Sex, male	52(59)	45(58)	0.529	18(49)	34(87)	< 0.001*
LVEDVi	44.55 ± 13.99	46.83 ± 13.98	0.301	36.53 ± 13.33	49.71 ± 17.77	< 0.001*
LVESVi	18.17 ± 8.21	19.70 ± 8.87	0.253	15.01 ± 6.83	26.96 ± 13.45	< 0.001*
LVMi, g/m2	88.5 ± 26.4	90.4 ± 22.1	0.605	99.3 ± 27.5	104.8 ± 27.6	0.393
LVEF, %	60 ± 9	58 ± 10	0.315	59 ± 8	47 ± 12	< 0.001*
GLS, %	−17.64 ± 3.81	−18.73 ± 3.99	0.077	−15.09 ± 4.38	−13.42 ± 3.82	0.080
LAVImin, ml	25.2 ± 11.4	21.6 ± 9.2	0.031*	44.0 ± 22.3	36.7 ± 11.1	0.071
LAVImax, ml	39.6 ± 14.7	34.0 ± 10.4	0.006*	55.2 ± 24.9	47.4 ± 13.1	0.090
Total emptying fraction	37.6 ± 13.5	37.2 ± 13.1	0.843	22.9 ± 10.2	22.8 ± 11.1	0.989
Function index	20.1(10.1–28.8)	24.2(14.1–40.3)	0.047*	6.7(4.1–9.0)	6.3(4.3–10.3)	0.089
Reservoir strain	21.00 ± 9.23	25.62 ± 9.54	0.002*	10.04 ± 5.87	12.63 ± 4.37	0.034*
Mechanical dispersion	56.7 ± 19.3	45.1 ± 13.7	< 0.001*	78.9 ± 25.9	52.6 ± 18.1	< 0.001*
Stiffness index	0.55(0.35–0.81)	0.39(0.31–0.62)	0.027*	1.23(0.97–1.82)	0.8(0.55–1.28)	< 0.001*
Ellipticity	88.4 ± 6.4	82.2 ± 5.7	< 0.001**	91.4 ± 5.3	89.3 ± 5.6	0.098
Circularity	91.1 ± 2.0	89.2 ± 2.2	< 0.001*	92.1 ± 1.8	90.8 ± 2.0	0.005*
Sphericity index	70.5 ± 13.4	55.1 ± 11.3	< 0.001*	76.9 ± 13.0	70.4 ± 13.0	0.032*
Eccentricity	86.7 ± 5.5	80.2 ± 5.5	< 0.001*	90.7 ± 5.6	88.7 ± 5.3	0.135

*P<0.05. AF: atrial fibrillation, *AF-NS*: atrial fibrillation with no prior stroke, *CES*: cardioembolic stroke, *EDVi*: end-diastolic volume indexed, *EF*: ejection fraction, *ESVi*: end-systolic volume indexed, *GLS*: global longitudinal strain, *LA*: left atrial, *LAVImax*: maximum left atrial volume indexed, *LAVImin*: minimum left atrial volume indexed, *LV*: left ventricular, *Mi*: mass indexed, *NS*: no stroke; *PAF*: paroxysmal atrial fibrillation, *perAF*: permanent atrial fibrillation, *TTE*: transthoracic echocardiogram

### PerAF TTE parameters

LV end-diastolic and end-systolic volumes were increased in AF-NS patients, (although within normal limits) (Table 4), with reduced LVEF compared to CES patients. Although no difference in LA volume was observed between groups, PerAF-CES patients had lower reservoir strain with increased mechanical dispersion and LA stiffness. CES patients had increased sphericity with higher circularity and sphericity index compared to AF-NS patients.

### Principle components analysis (PCA)

Plausible variables were determined by sensitivity analysis (Supplementary Data, Table S3) and collinearity assessment and included circularity, emptying fraction, minimum and maximum indexed volumes, and stiffness index. Variables were scored with eigenvalues and combined in a series of latent factors (Table 5) for PCA model distribution of AF groups (Fig. 5):

**Table 5:** PCA analysis of LA parameters in patients with varying grades of AF

	PCA1		PCA2	
	LF1	LF2	LF3	LF4
Circularity	0.54	0.80	0.53	0.79
Total emptying fraction	−0.72	0.23	−0.69	0.42
LAVImin	0.95	−0.09	0.95	−0.13
LAVImax	0.87	0.05	0.87	0.09
Stiffness index	0.71	−0.32	0.70	−0.12
Variance explained	58%	14%	55%	14%

PCA1: CES patients

PCA2: CES patients and patients with NS-AF

*AF*: atrial fibrillation, *CES*: cardioembolic stroke, *LA*: left atrial, LAVImax: maximum left atrial volume indexed, *LAVImin*: minimum left atrial volume indexed, *LF*: latent factor, *PCA*: Principal component analysis

PCA 1: AF discrimination in CES patients – latent factors (LF1, LF2).

PCA 2: AF discrimination in CES and NS-AF patients – latent factors (LF3, LF4).

Scatterplots of PCA1 scores demonstrated ability to differentiate AF groups; LF1 and LF2 resolved 76% of the overall variance between CES patients with different levels of AF (LF1 60%, LF2 16%). Inspection of PCA1 score-plot (Fig. 5a) demonstrates patients in sinus rhythm are generally clustered to the left with negative LF1. In contrast, patients with perAF have high positive LF1, indicating higher values of sphericity, volume, stiffness, and reduced LA function. PAF are observed mid-way with some overlap with other groups (both positive and negative values of LF1), and are clustered with higher LF2 values, indicating increased LA sphericity with reduced LA stiffness.

Comparison of CES patients to NS-AF patients in PCA2 (Fig. 5b) also had good differentiation using LA variables, with 75% overall explanation of variance (LF3 58%, LF4 17%). NS-AF patient clusters had smaller dispersion than rhythm-matched CES patients, indicating stroke patients have larger LA volume, stiffness and circularity, with reduced LA function notwithstanding level of AF. CES patients in sinus rhythm had low LF3 with patients in perAF trending high, and PAF patients centred between both groups. NS-AF patients with PAF and perAF show a similar LF3 distribution, however trending slightly lower to corresponding CES patients with matched AF grade. Interestingly, LF4 distribution is slightly higher in PAF patients with stroke compared to PAF patients without stroke, indicating a subset of CES patients with increase in sphericity and function compared with NS-AF patients.

### ROC analysis

**Fig. 6:**
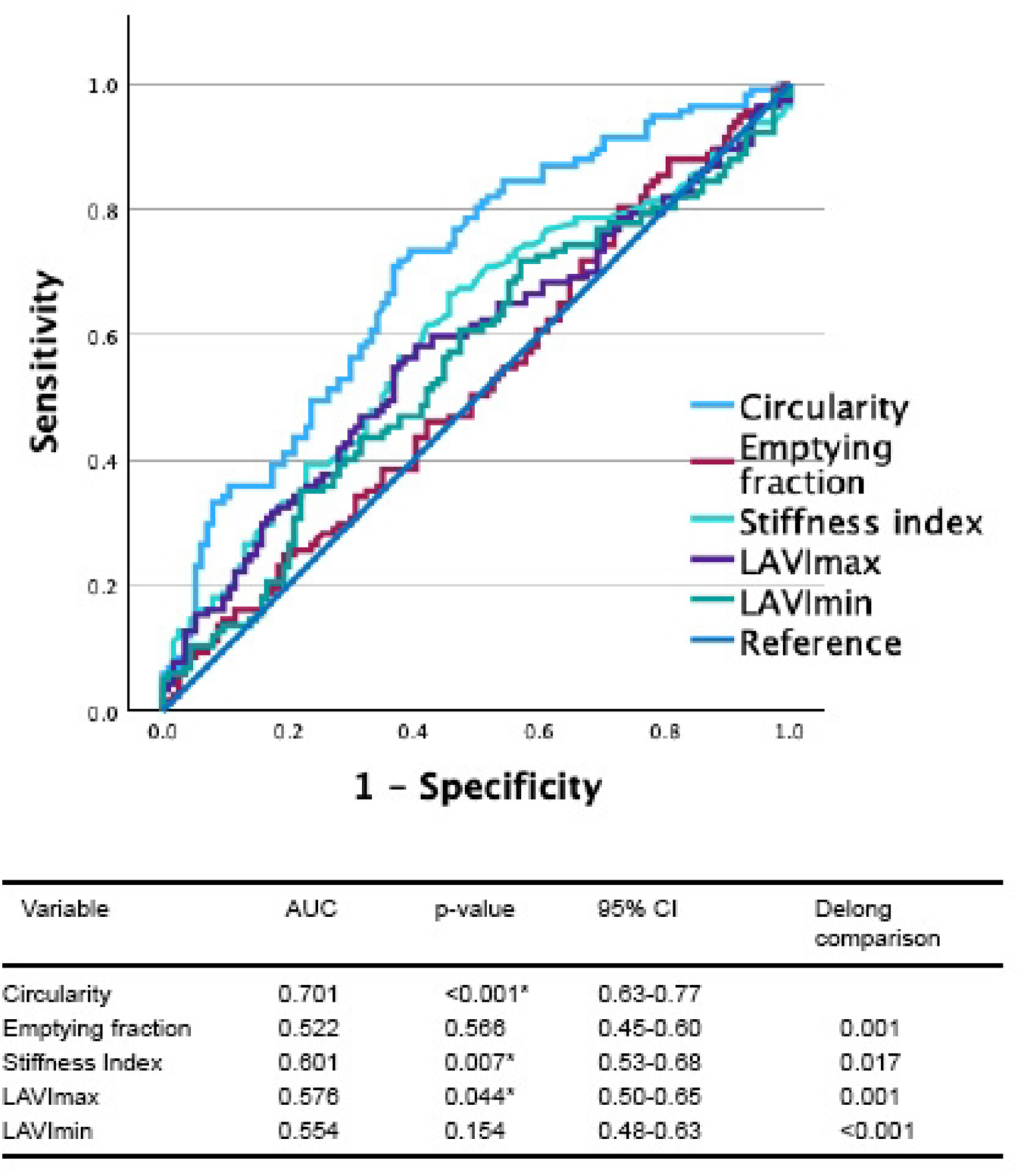
ROC curve analysis for LA parameters to predict stroke in patients with AF. Largest AUC is observed for circularity and mechanical dispersion. AUC: area under the curve, CI: confidence interval, LA: left atrial, LAVImax: maximum indexed LA volume LAVImin: minimum indexed LA volume

We additionally performed ROC analysis to futher assess the discrimination of CES, using the plausible LA parameters identified by PCA assessment (Fig. 6). Circularity had the highest AUC (0.701, *p* < 0.001) followed by LA stiffness index (AUC 0.601, *p* = 0.007) and LAVImax (AUC 0.576, *p* = 0.045). LAVImin and LA emptying fraction did not demonstrate significant ability to discriminate stroke. Delong pairwise comparison showed the AUC for circularity was significantly higher than all other parameters.

### Reproducibility

A high degree of intra- and interrater reproducibility was observed for ICC and CV measures of volume, strain, and sphericity (Supplementary Data, Table S4).

## Discussion

In this prospective study of CES patients we performed comprehensive evaluation of traditional and novel LA parameters. As expected, we found significant alterations in traditional LA paramaters, with increased LA volume and reduced function observed with increased level of AF. Significant alteration was also observed in novel measures of stiffness and sphericity: Both parameters were found to increase with increased level of AF. Further, we established variation in LA measurements of CES patients with AF, when compared to AF-NS patients. Notably, PCA analysis of latent factors, comprising a comprehensive set of LA parameters, enabled both differentiation of CES patients based on rhythm, and CES patients from AF-NS patients. Further, PCA demonstrated the most influential LA parameters required for discrimination include LA volume, function and the novel measures of sphericity and stiffness.

A novel aspect of this prospective study is that the methodology employed a detailed evalution of left atrial characteristics using traditional (LA volume and function), and less well established and emerging measures of LA stiffness and sphericity. These findings substantiate previous findings of LA remodelling in stroke [9] and adverse cardiac outcomes in AF patients [24]. Furthermore, there were differences in patients with AF without a stroke (AF-NS) versus those who had CES due to AF, suggesting that novel LA parameters may help stratify cardioembolic stroke risk in patients with known AF. However, CES-AF patients had more cardiovascular risk factors, which could in part, result in the differences observed in LA parameters.

We examined different measurements of sphericity, and while all the measures demonstrated a significant increase in sphericity as grade of AF increased, sensitivity analysis determined ‘circularity’ as the most plausible sphericity variable for model prediction. We surmise that the use of area and circumference for geometric calculation of circularity may provide a more robust measure than the other methods where formulae are reliant on Linear dimensions, particularly in AF. Circularity, while demonstrating the highest AUC of 0.7, is only a modest discriminator for the identification of CES patients from AF-NS, but had the highest AUC compared to other LA parameters (volume and function). Our findings from this pilot study suggests that LA sphericity may be a significant discriminator of LA remodelling, but requires validation studies.

Increasing LA sphericity may alter LA blood flow (flow being one of the components of Virchow triad), thereby promoting thromboembolism. LA sphericity ascertained by computed tomography has previously demonstrated correlation with thromboembolic events in AF patients [25] and increased AF recurrence following ablation [26].

Sphericity is also associated with LA fibrosis [27], reduced LA reservoir strain, and increased LA pressure in AF patients [28]. Spherical versus ellipsoid phantom modelling have demonstrated reduced flow velocity with associated coagulation cascade activation [29], while intracardiac areas of fluid turbulence and reduced vortex formation correlate with reduced endocardial shear stress, enabling platelet adhesion and aggregation [30].

Reduced LA strain is known to precede LA dilatation and to predict incident AF [31]. LA strain is significantly reduced in AF [32], and demonstrated significant reduction in CES patients versus rhythm matched NS-AF patients. However, on PCA, LA strain was not identified as a parameter useful for CES risk stratification in various AF grades, compared to other LA parameters. We surmise this may be analogous to the increased sensitivity of global longitudinal strain in detection of subclinical LV dysfunction when LV ejection fraction is relatively preserved, prior to myocyte stretch and ventricular remodelling [33]. Similarly LA strain may have discriminative value in early LA dysfunction rather than in a remodelled atrium as noted in patients with AF.

AF has been considered instrumental in thrombus formation in CES, with a 5-fold increase in stroke risk [3]. The exact mechanism of thrombogenesis remains complex with an indistinct temporal relationship between AF episodes and stroke. AF involves structural, functional and electrical remodelling [34] with thrombogenesis posited to occur due to low LA flow velocities and regional stasis, as demonstrated by 4D-flow CMR [29, 35]. Additionally, flow within the LA may be altered based on LA sphericity, and when combined with AF increase the risk of thrombogenesis. However, AF may often be undetected till presentation with stroke, especially in patients with PAF and hence result in a low percentage of PAF patients receiving anticoagulation, as demonstrated in our cohort (17% in PAF versus 60% in Per AF).

As expected we found LA enlargement and dysfunction with worsening AF grades, consistent with previous findings [36]. Additionally, we demonstrate that novel measures of LA stiffness and sphericity may further characterise LA alterations, with increased LA stiffness and circularity observed with worsening grades of AF, and enhancing discrimination between CES and AF-NS cohorts. Increasing LA stiffness likely occurs following fibrotic replacement of atrial myocytes, and could result from increased LV filling pressure [37]. Spherical remodelling of the left atrium is also plausibly compensatory, representing structural efficiency of a thin-walled chamber from increased hydrostatic pressure [38].

### Limitations

There are several limitations in this study. This was a single-centre exploratory study, with modest patient cohorts. We were unable to age-match CES groups, with patients in sinus rhythm being younger. However, reduced LA reservoir strain was observed in these patients, and highlights early atrial functional remodelling in this group. This was a cross-sectional study, hence the predictive value and clinical utility of LA parameters in AF and CES prevention could not be determined. Longitudinal studies, incorporating LA stiffness and sphericity in AF/CES risk-prediction models for stroke recurrence will provide such information. Our preliminary findings need to be evaluated in larger prospective studies to determine their role in CES risk-stratification. 2D TTE assessment of stiffness and sphericity would benefit from comparison to MRI and 3D TTE-derived parameters to strengthen measurement validity, while 4D atrial flow may elucidate atrial flow dynamics and vortices consequent to altered LA sphericity. Finally, in this study we did not include comparisons of CES-AF with non-cardioembolic stroke patients to examine if alterations in LA characteristics would be observed in this group as well, in order to better ascertain the specific role of AF in CES.

## Conclusions

We have performed comprehensive echocardiographic assessment of the left atrium and demonstrated LA remodelling in patients with CES when stratified by rhythm. Additionally, deep phenotyping of novel LA parameters demonstrated differences between CES patients and patients with AF and no prior history of stroke. We demonstrate that LA remodelling in CES patients involves complex alterations of a variety of LA parameters, including novel parameters of LA stiffness and sphericity, in addition to established measures of LA volume and function. We speculate that a combination of LA parameters could improve stroke risk prediction in AF patients, with higher prognostic value than just LA volume and function. Future prospective studies that incorporate these novel LA parameters into AF and stroke risk prediction models are needed to validate their utility.

## Supplementary Information

Below is the link to the electronic supplementary material.

Supplementary Material 1

## Data Availability

No datasets were generated or analysed during the current study.
