# Supplementary material for: Deep phenotyping of left atrial characteristics: sphericity and stiffness stratify patients with atrial fibrillation and cardioembolic stroke
Source: Int J Cardiovasc Imaging. 2025 Sep 18;41(11):2097–111. doi: 10.1007/s10554-025-03510-x (PMC12628483; doi:10.1007/s10554-025-03510-x)
Supplement: Supplementary file 1 — Supplementary Material 1 [file 10554_2025_3510_MOESM1_ESM.docx]

**Supplementary Table S1:**

**Comprehensive echocardiogram measurements and calculations**

| **Parameter** | **Method and Calculation** |
| --- | --- |
| LV linear dimensions^1^ | Parasternal Window, PLAX view.  Callipers were perpendicular to LV long axis with measurement of IVS, LVID, and PW at end-diastole. |
| LV mass (indexed)^1^ | Cube formula:  = 0.8 · 1.04 · [(IVS + LVID + PW)3 −LVID3] + 0.6g  Mass was indexed to BSA (i) |
| LV end-diastolic (EDV) and end-systolic (ESV) volumes (indexed)^1^ | Apical window, biplane apical 4- and 2-chamber views.  LV cavity blood-tissue interface was traced at end-diastole ad end-systole. Biplane method of disk summation (modified Simpson’s rule) was used to calculate volume.  Volumes were indexed (i) to BSA. |
| LV ejection fraction^1^ | = (EDV − ESV) / EDV. |
| LVOT VTI^2^ | Apical window, 5-chamber view.  Pulsed-wave Doppler with sample volume proximal to aortic valve. Signal was traced for VTI calculation. |
| Mitral inflow^2^ | Apical window, 4-chamber view.  Pulsed-wave Doppler with sample volume placed at the tips of open mitral valve. Peak early (E) and atrial contraction (A) velocities, and early diastolic deceleration time were measured, with calculation of E/A ratio. |
| Mitral e’^2^ | Apical window, 4-chamber view.  Doppler Tissue Imaging (DTI) of medical and lateral mitral annulus. Peak early diastolic (e’) velocity was measured at each point and averaged. |
| E/e’^2^ | Ratio of peak early diastolic mitral inflow velocity (E) to averaged early diastolic mitral annulus tissue velocity (e’). |
| LV GLS^2^ | Apical window, 2-, 3-, and 4-chamber views.  2D speckle-tracking of an 18-segment model with endocardial border tracking. Peak systolic strain was measured from each view to calculate global longitudinal strain (GLS). |

**Supplementary Table S2:**

**Source of cardioembolic stroke in sinus rhythm**

| **Source of cardioembolic stroke in sinus rhythm** | **Number of patients** |
| --- | --- |
| Patent foramen ovale with no other identifiable cause | 20 (66.7) |
| Left ventricular impairment | 5 (16.7) |
| Endocarditis | 1 (3.3) |
| Myxoma | 1(3.3) |
| Idiopathic ventricular tachycardia | 1 (3.3) |
| Left atrial thrombus without atrial fibrillation | 1 (3.3) |
| >1 vascular territory with history of myocardial infarction and uncontrolled risk factors | 1 (3.3) |

**Supplementary Table S3:**

**Multivariate analysis of left atrial measures**

| **Group** | **Variable** | **Univariable Likelihood Ratio Test** | **Multivariable Likelihood Ratio Test** |
| --- | --- | --- | --- |
| **Left atrial sphericity** | LA Ellip | <0.01 | 0.82 |
|  | LA Circ | <0.01 | 0.04 |
|  | LA spher.i | <0.01 | 0.22 |
|  | LA eccent.i 4Ch | <0.01 | 0.85 |
|  | LA eccent.i biplane | <0.01 | 0.83 |
| **Left atrial functionality** | LAFI | <0.01 | 0.32 |
|  | LASr | <0.01 | 0.26 |
|  | LAEF | <0.01 | 0.01 |
| **Left atrial volume** | LAVImin | <0.01 | <0.01 |
|  | LAVI max | <0.01 | <0.01 |
| **Left atrial dyssynchrony** | LAMD | <0.01 | 0.11 |
|  | LAMD% | <0.01 | 0.81 |
| **Left atrial stiffness** | LASI | <0.01 | -- |

**Supplementary Table S4:**

**ICC and CV for intra- and interobserver variability for TTE assessment of left atrial parameters**

|  | **Intraobserver** | | **Interobserver** | |
| --- | --- | --- | --- | --- |
| **Variable** | **ICC variability** | **CV %** | **ICC variability** | **CV %** |
| **LAVImin** | 0.99(0.98–1.00) | 8.0 | 0.97(0.93–0.99) | 10.1 |
| **LAVImax** | 0.95(0.85–0.98) | 5.8 | 0.95(0.86–0.98) | 8.2 |
| **Reservoir strain** | 0.99(0.97–1.0) | 7.6 | 0.99(0.98–1.0) | 8.4 |
| **Ellipticity** | 0.90(0.74–0.97) | 2.6 | 0.94(0.83–0.98) | 6.3 |
| **Circularity** | 0.91(0.74–0.97) | 1.0 | 0.95(0.85–0.98) | 2.4 |
| **Sphericity Index** | 0.91(0.75–0.97) | 5.9 | 0.93(0.81–0.98) | 7.1 |
| **Eccentricity** | 0.95(0.85–0.98) | 2.4 | 0.91(0.74–0.97) | 5.9 |

Abbreviations: CV: coefficient of variation, ICC: intraclass correlation coefficient, LAVImax: maximum left atrial volume indexed, LAVImin: minimum left atrial volume indexed, TTE: transthoracic echocardiogram

**Supplementary References**

1. Lang RM, Badano LP, Mor-Avi V, Afilalo J, Armstrong A, Ernande L, et al. Recommendations for cardiac chamber quantification by echocardiography in adults: an update from the American Society of Echocardiography and the European Association of Cardiovascular Imaging. Eur heart j cardiovasc imaging. 2015;16(3):233-271.

2. Mitchell C, Rahko PS, Blauwet LA, Canaday B, Finstuen JA, Foster MC, et al. Guidelines for Performing a Comprehensive Transthoracic Echocardiographic Examination in Adults: Recommendations from the American Society of Echocardiography. J Am Soc Echocardiogr. 2019;32(1):1-64.
